# The effectiveness of educational interventions in the community that aim to improve informal carers knowledge of dementia anatomy, physiology, progression, and impact on behavior: a systematic review

**DOI:** 10.3389/frdem.2023.1156863

**Published:** 2023-06-27

**Authors:** Dayna Bushell, Cindy Jones, Christian Moro

**Affiliations:** ^1^Faculty of Health Sciences and Medicine, Bond University, Gold Coast, QLD, Australia; ^2^Menzies Health Institute Queensland, Griffith University, Nathan, QLD, Australia

**Keywords:** dementia education, neuroanatomy, neurophysiology, informal carers, patient education

## Abstract

**Introduction:**

Dementia education is a vital component of dementia care and management for patients and their informal carers and family. To fully understand dementia, some knowledge of the anatomy and physiology of the brain may be necessary and would help informal carers understand behaviors of dementia to help cope with care provision.

**Method:**

This integrative review aims to identify, appraise, and assess whether dementia education resources include information detailing the anatomy of the brain and its relationship with dementia and whether this information improves knowledge (PROSPERO Registration Number: CRD42022320530). Literature published from 2012 until May 4, 2022 was searched in eight databases with six articles meeting the inclusion criteria.

**Results:**

Using the Mixed Methods Appraisal Tool (2018) methodological quality varied across studies. There are limited educational interventions which incorporate information on the anatomy and the physiology of the brain. None of the interventions focused solely on providing neurological education; however, all contained at least some content that addressed this, as per inclusion criteria. In most cases, the educational interventions were well-received and delivered, which did not differ, whether they were delivered in person or virtually. The majority of the studies reported an increase in dementia knowledge (measured pre-post or perceived) following the intervention.

**Discussion:**

Educational interventions on brain anatomy and physiology remain limited, and if included, are often not the focus, and as such more rigorous study is required to investigate the effect of educational interventions on dementia knowledge and their role in dementia care.

## 1. Introduction

**Figure 1:**
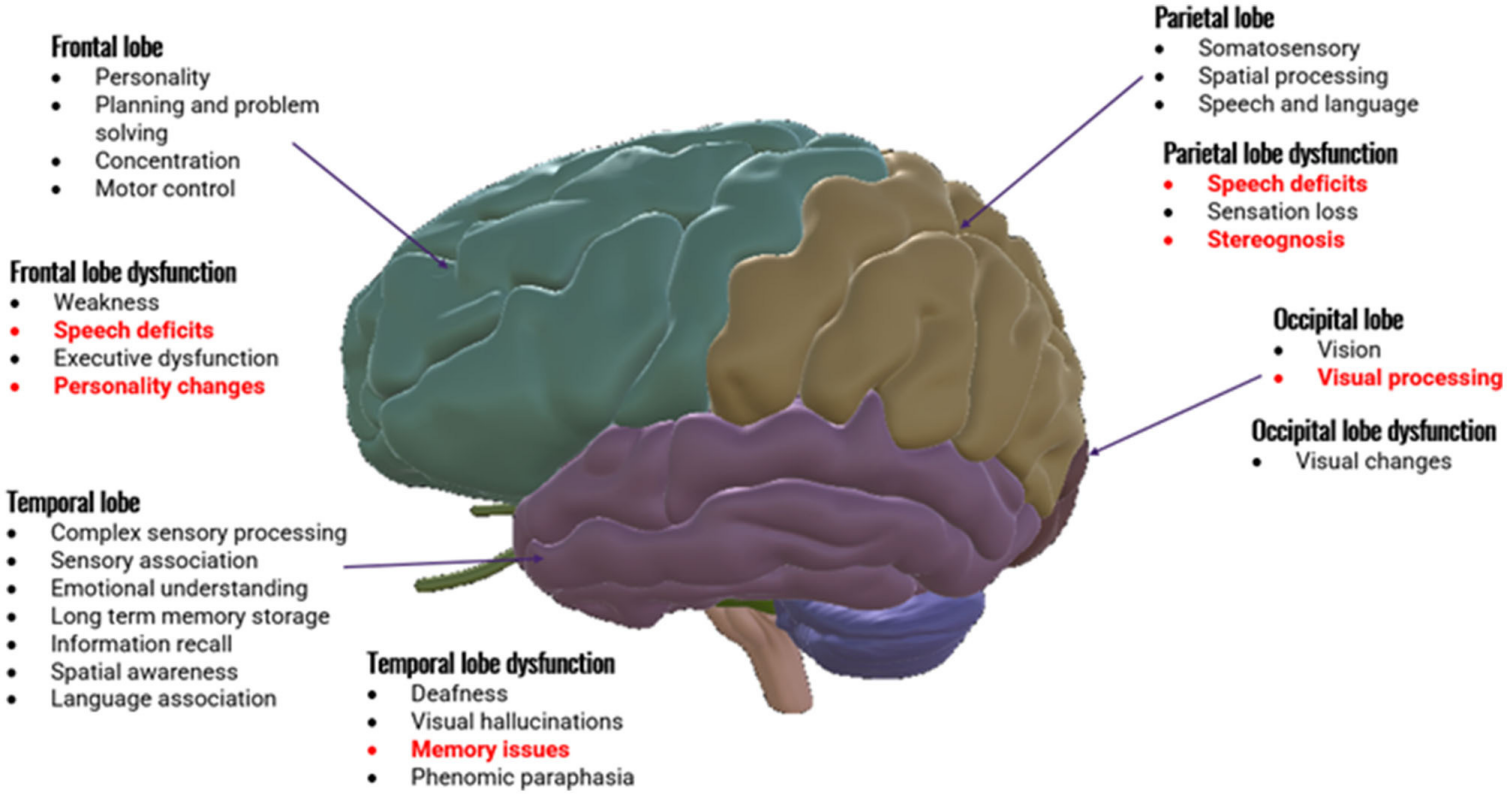
Anatomy of brain divided into cerebral lobes with normal functions of each lobe. Generalized dysfunctions are outlined. Specific symptom presentations common in Alzheimer's disease with the commonly affected lobes are indicated in red.

Dementia is a complex clinical syndrome characterized by gradual, persistent, and progressive cognitive decline, which interferes with the patient's ability to function independently. Cognitive impairments are due to damage to the cerebral cortex resulting from synaptic failure, inflammation, cerebral metabolism changes, and neuronal death (Savva et al., 2009; Cunningham et al., 2015; Hildreth and Church, 2015). The clinical presentations of dementia vary, based on the underlying pathophysiology and region of brain affected and can present as memory loss, communication impairments, apraxia, and impaired executive function (McKhann et al., 2011; Cunningham et al., 2015; Duong et al., 2017). Figure 1 below shows an image of the brain with the normal functions for each lobe outlined. Common symptom presentations in Alzheimer's disease, corresponding with specific anatomical areas, are indicated in red.

As of 2020, there were ~55 million people living with dementia worldwide. These figures are expected to increase to 139 million by 2050, with an estimated 10 million new cases every year (World Health Organisation, 2022).

The World Health Organization has identified a lack of awareness and understanding of dementia, leading to stigmatization and barriers in diagnosis and care. As such, dementia education sits as one of the seven cross-cutting principles outlined within the World Health Organization's global action plan on the public health response to dementia. Some key action areas within this plan include improving dementia awareness and friendliness, increasing dementia information systems, research, and innovation, and enhancing support for dementia carers. These action areas involve developing dementia-awareness programs to foster an accurate understanding of dementia and reduce stigmatization for both the general community and informal carers (i.e., including families) of people living with dementia (World Health Organisation, 2017).

Responsive behaviors associated with dementia, or also known as behavioral and psychological symptoms of dementia, are common and can cause distress to the patient and carers (Duong et al., 2017). In 2019, informal carers spent an average of 5 h per day providing care to people with dementia. This caregiving can result in significant physical, emotional and financial distress (World Health Organisation, 2022). Contributing to the distress is a lack of accessible, detailed information surrounding the dementia diagnoses available for carers and patients (Robinson et al., 2009). Engaging informal carers in the diagnosis and treatment process is crucial (Wesson and Reitman, 2012; Molnar and Frank, 2018), as they are involved in multiple areas of care (Khanassov et al., 2021). However, these informal carers often report a large number of unmet needs (Zwingmann et al., 2019; Khanassov et al., 2021), with 90% of carers identifying education and information as an important issue (Kurz et al., 2008). Informal carers and people with dementia reported receiving insufficient information from primary healthcare providers, leading to a lack of understanding surrounding the diagnosis (Peterson et al., 2016; Khanassov et al., 2021). Evidence suggests that little time is spent explaining a dementia diagnosis resulting in poor retention rates whereby 70.3% of people living with dementia and 16.2% of family members were unable to accurately report the diagnosis following a clinical consultation (Barrett et al., 2006). This confusion and uncertainty can lead to difficulty in prognosis discussions and future planning. One study that analyzed patients ability to correctly report amyloid-β PET scan results for patients with mild cognitive impairment (MCI) or dementia found that even when patients can report the result, they are often unsure about the meaning. The authors suggested that increased provider information, information, and education is needed to improve this understanding (James et al., 2020).

Generally, dementia education for informal carers has primarily focused on caregiving skills and strategies to improve caregivers mental and physical health (Cheng and Zhang, 2020). However, it remains unclear whether existing educational interventions include information on the disease mechanisms in dementia and its influence on the anatomy and physiology of the brain, which can lead to improvements in health literacy and is vital to comprehending the disease and its progression. This particular area of education is important as it allows informal carers to interact more effectively with the healthcare systems and make well-informed decisions surrounding treatment and care (Lee et al., 2004; Eccleston et al., 2019). It can also increase informal carers' understanding of the impacts of dementia on behaviors and daily activities of living, and the appropriate selection of care management approaches, potentially leading to better care provision and outcomes for both the person with dementia and the informal carers. Additionally, it may also improve carer wellbeing which can assist with keeping people with dementia living in the community, rather than being placed in long-term care (Mittelman et al., 2006). A lack of understanding of the behavioral changes from dementia leads to difficulties in informal carers distinguishing the person with dementia from the disease, with their behaviors being attributed to “bad” or “negative” rather than as characteristics of dementia (Tarrier et al., 2002; Polk, 2005). These attributions can cause higher care burden and elevated depression as the symptoms are believed to be controllable, personality traits, rather than symptoms of dementia (Tarrier et al., 2002; Williamson et al., 2005; Polenick and Martire, 2013; Polenick et al., 2018). A study surrounding attributions and dementia BPSD was conducted analyzing 26 family carers in four focus groups. This study found that while some informal carers knew that brain changes were occurring, not all able to identify what these changes involved with one participant explaining the change by stating that “*the disease is causing wiring problems in the brain”*. Other participants identified other medical conditions, environmental triggers, and psychological feelings as some of the causes of BPSD. When attributing the controllability of BPSD some caregivers indicated that they believed that the symptoms were partly controllable and referenced pre-diagnoses personality characteristics. Other informal carers stated that they believed the person with dementia had deliberate or malicious intent or were voluntarily displaying BPSD around certain people (Polenick et al., 2018). Therefore, while some informal carers understand that dementia is a brain disease, the attribution of symptom controllability highlights the need for further education and information surrounding the brain changes and how this affects behavior in order to assist with differentiation between willful and involuntary behaviors. As such, it is crucial to design, evaluate and implement effective and accessible dementia education for informal carers of people with dementia.

## 2. Methods

### 2.1. Review aim and questions

The aim of this integrative review is to identify, appraise, and synthesize the existing evidence on educational interventions for informal carers of people with dementia to answer the following questions:

What effect do educational interventions, which covers anatomy and physiology of the brain, have on improving the dementia-specific knowledge of informal carers of people with dementia?

What is the learning experience of informal carers who utilize these educational interventions?

### 2.2. Protocol

#### 2.2.1. A priori protocol

The Preferred Reporting Items for Systematic Reviews and Meta-Analyses (PRISMA) (Page et al., 2021) was followed in this review (Supplementary B). The protocol was developed prior to the beginning of the review and registered by PROSPERO on 24 March 2022 (CRD42022320530). The submitted protocol was followed with no deviations.

#### 2.2.2. Design

This review used an integrative approach to assess study outcomes from various methodologies (i.e., quantitative, qualitative, and mixed methods) and data types in order to establish a comprehensive understanding and answer the review questions. This approach was used to ensure that all relevant studies were incorporated and assessed to contribute to the knowledge and understanding of current educational interventions for people with dementia and their informal carers.

#### 2.2.3. Search strategy

Eight electronic databases covering health, science, psychology, medicine, and education were searched. These included CINAHL (EBSCO), MEDLINE (Ovid), Web of Science Core Collection, Cochrane Central Register of Controlled Trials (CENTRAL; Wiley), Embase (Elsevier), PsycINFO (Ovid), ERIC (Ovid) and Scopus. Articles were identified using the following search strategy: (dementia:ti,ab OR alzheimer^*^:ti,ab OR 'dementia'/exp) AND (education^*^:ti,ab OR program^*^:ti,ab OR 'e learning':ti,ab) AND (progression:ti,ab OR behav^*^:ti,ab OR knowledge:ti,ab OR 'disease exacerbation'/exp) AND (caregiver^*^:ti,ab OR carer^*^:ti,ab OR famil^*^:ti,ab OR 'carers'/exp). The Polyglot search tool (Clark et al., 2020) was used to translate and check the title, abstract and keyword search terms for all databases. Complete search strategies for all databases are presented in Supplementary A. A forwards-backwards scan was performed, incorporating the reference lists of included articles to identify any other publications; however, none were identified.

#### 2.2.4. Inclusion/exclusion criteria

To be included in this review, articles had to meet the following inclusion criteria: (i) published in English within the last 10 years (January 2012 to May 2022); (ii) conducted in Australia or in countries with similar healthcare system (e.g., United States of America, United Kingdom, Canada, etc.); (iii) report original data using quantitative, qualitative, or mixed methods approaches and (iv) focus on dementia education for people diagnosed with dementia living in the community, their family and/or informal carers. Studies must also include outcomes on knowledge and education on brain anatomy and physiology to be considered for inclusion. Articles were excluded that only reported: (i) education for professionals or formal carers, people with dementia living in long-term care facilities, family members of people with dementia in long-term care, medical students or health professionals learning about dementia; (ii) trial registration materials; (iii) without full-text report; (iv) non-original research such as review articles, newsletters, editorials, opinion papers, dissertations, commentaries, discussions document and press reports; (v) studies focused on patient experiences or solely aiming to change attitudes (not including knowledge gained on the disorder's progression or trajectory). Conference abstracts were also excluded, with the exception of full-text, peer-reviewed conference papers. Articles focused solely around educating on detecting early signs of dementia were also excluded.

#### 2.2.5. Search outcome

**Figure 2:**
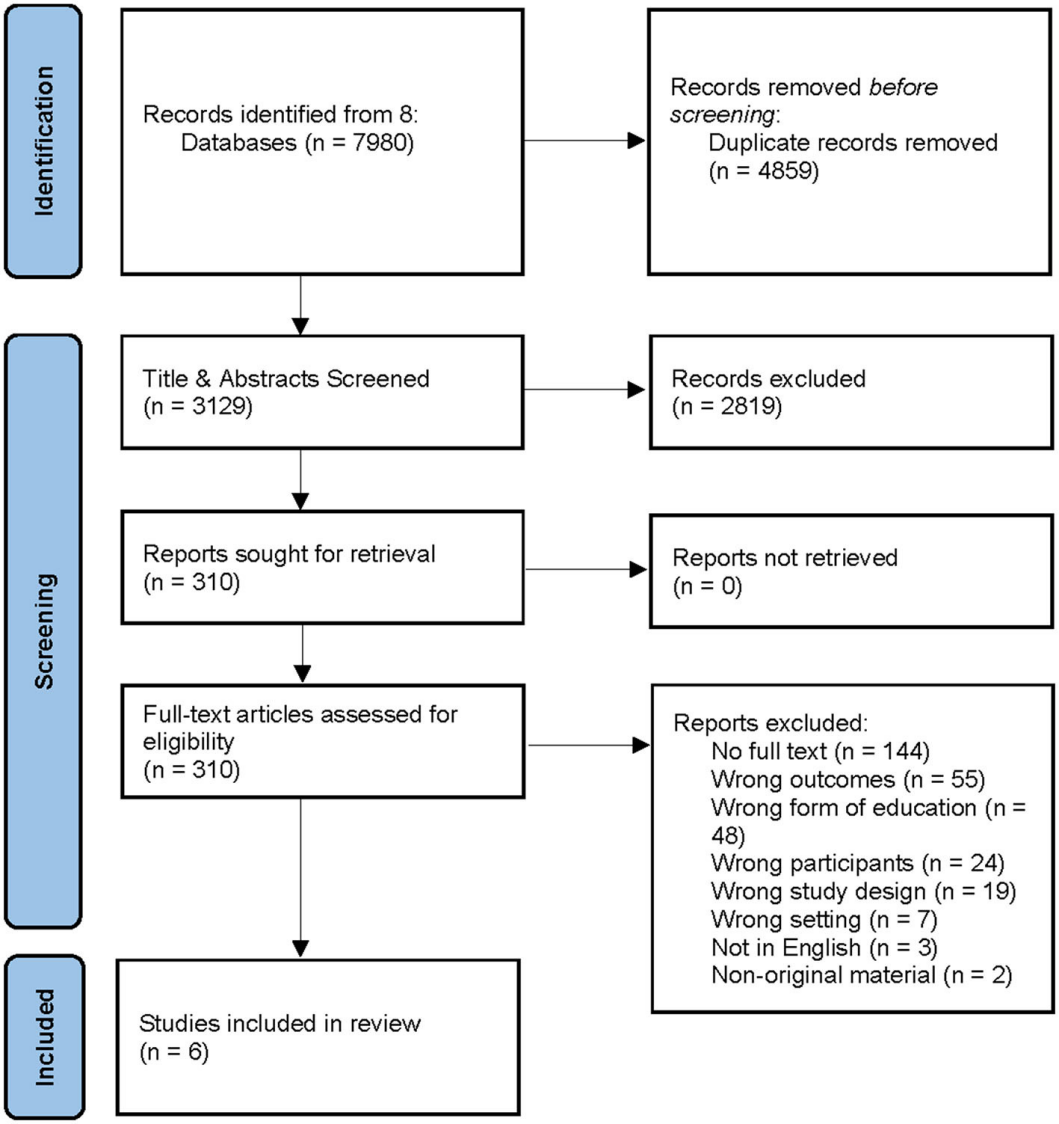
Integrative review flow chart: study selection process.

After removing any duplicates from the search results, a screening of the retrieved titles and abstracts was independently conducted by two authors (DB & CM) according to the inclusion and exclusion criteria. The full texts of eligible studies were then retrieved and assessed by two authors (DB & CJ). Any disagreements were resolved via discussion with the third author (CM). Six articles were included in the review (Figure 2).

#### 2.2.6. Quality appraisal

Two review authors (DB & CJ) independently assessed the quality of included studies using the Mixed Methods Appraisal Tool Version 2018 (MMAT2018) (Hong et al., 2018). Disagreements were resolved through discussion with a third reviewer (CM). The MMAT includes two initial questions which screen for empirical studies. Studies are not suitable for inclusion if they do not satisfy these initial questions. Following this, studies can be appraised based on study design (e.g., quantitative randomized and non-randomized controlled studies, quantitative descriptive studies, qualitative studies, and mixed-methods studies) using core quality criteria with the outcome reported descriptively for each criterion.

#### 2.2.7. Quality extraction and analysis

The following data was extracted from the studies into a table using Covidence: author name, country of publication, aim of study, study design, population and sample size, education intervention details, outcome measurement tool(s), key outcomes, and quality appraisal. This process was done independently (DB & CJ) and cross-checked. Any disagreements were resolved by consensus or by the third author (CM). Requests were to be made to appropriate original authors to provide any missing data, although this was not necessary. An integrative review involving qualitative, quantitative, and mixed-method studies was used. A narrative (descriptive) synthesis of the findings, which involves the reduction, display and comparison of data followed by the drawing and verification of conclusions, was used to synthesize data from all included studies as per Whittemore and Knafl (2005). Subgroup analysis was performed, where appropriate, based on participants' type (person with dementia or family/informal carers) which the intervention was applied to, in order to determine the effectiveness of educational interventions in each subgroup. Meta-analysis was not performed due to the heterogeneity of included studies.

## 3. Results

### 3.1. Study characteristics

The earliest paper, of the six included articles, was published in 2015 (Hattink et al., 2015), with the most recent published in 2022 (Noel et al., 2022). Two studies were conducted in the USA (Easom et al., 2020; Noel et al., 2022), one study was conducted simultaneously in both the Netherlands and the United Kingdom (Hattink et al., 2015), one solely in the Netherlands (van Wezel et al., 2021), one conducted by an Australian research team, but available for participants globally (Eccleston et al., 2019), and one solely in Australia (Taylor-Rubin et al., 2020). Two studies were quantitative randomized control trials (RCT) (Hattink et al., 2015; van Wezel et al., 2021), three were quantitative non-RCT studies (Eccleston et al., 2019; Easom et al., 2020; Noel et al., 2022), and one was a mixed-methods study (Taylor-Rubin et al., 2020). Table 1 shows a summary of the selected articles and quality assessment, and Supplementary C displays the full quality assessment using the MMAT2018.

**Table 1:** Summary of the selected articles.

**References and country**	**Study aim**	**Study design**	**Setting and population**	**Intervention**	Outcomes^*^	Key findings^*^	**Study Quality (MMAT2018)**
van Wezel et al. (2021), Netherlands	To examine the effects of the educational peer-group intervention on knowledge about dementia, perceived ability to talk about it, received support and self-perceived pressure from informal care among family carers with a Turkish or Moroccan immigrant background who cared for a person with dementia.	Randomized controlled trial	Community center. Turkish or Moroccan family (*n =* 550). - Intervention (*n =* 288) - Control (*n =* 262)	Two educational sessions. Session 1: Informational (difference between dementia and normal forgetfulness, explanation of dementia as a brain disease). Session 2: group discussion.	Knowledge about dementia (ADKS), perceived ability to talk about dementia (Likert scale)	- Significant trend of increased knowledge over time - Significantly stronger increase for intervention condition (p < 0.001).	- Incomplete outcome data - Unable to tell if outcome assessors were blinded
Taylor-Rubin et al. (2020), Australia	Evaluate the benefits of a 3-hour PPA-specific group education and support session for pwPPA and their carers	Pre-post intervention study	Clinic. People with primary progressive aphasia and carers of people with primary progressive aphasia (*n =* 25) - pwPPA (*n =* 12) - Carers (*n =* 13)	3-hour PPA-specific education program with 4 components: disease education, psychoeducational and practical strategies for managing stress, worry, and low mood, practical strategies for maximizing communication, and peer support.	Knowledge of PPA (5-point Likert scale)	- Significant increase for first session pwPPA carers (p < 0.01) and second/subsequent pwPPA carers (p < 0.025). - All first session pwPPA (*n =* 4) increased knowledge rating - Little change in knowledge for second/subsequent pwPPA. - Qualitative theme: Improved understanding of PPA.	- Incomplete outcome data - Unable to tell whether confounders are accounted for
Hattink et al. (2015), Netherlands and United Kingdom	The objective of the current study was to evaluate the user friendliness, usefulness, and impact of STAR with informal carers, volunteers, and professional carers.	Randomized controlled trial	Virtual. Participants caring for someone with dementia as an informal carer or a volunteer in dementia care, living in the Netherlands or United Kingdom (*n =* 142) - Laypeople (informal carers or volunteers) (*n =* 59) - Intervention (*n =* 27) - Control (*n =* 32)	STAR Training Portal: 8 modules (what is dementia, living with dementia, getting a diagnosis and why it is important, practical difficulties in daily life, emotional impact of dementia, support strategies, positive and empathic communication, emotional impact and looking after yourself).	User friendliness, usefulness (USE), and impact of STAR on knowledge (ADKS)	- No significant difference in pre- and post-test scores for ADKS (p = 0.90) - Positive about the usefulness of the different modules. - High usefulness and high user-friendliness of most elements.	- Incomplete outcome data - Outcome assessors not blinded
Eccleston et al. (2019), Australia (delivered globally)	To assess the effectiveness of the UDMOOC in educating people about dementia in a broad international community by assessing knowledge in a pre-post design over 2 years	Pre-post intervention study	Virtual. All users of UDMOOC (*n =* 27,265) - UDMOOC participants (*n =* 8498) and DKAS participants (*n =* 1638) were family members of someone diagnosed with dementia. - UDMOOC participants (*n =* 718) and DKAS participants (*n =* 137) were unpaid carers.	Understanding Dementia Massive open online course. 9-week program focusing on basic neurobiology, dementia pathophysiology, medical management, and person-centered care.	Knowledge of dementia (DKAS)	- Median baseline scores: 34.5/50 (IQR 27-41). - Median Post-UDMOOC score: 45 (IQR 41-48). - All groups showed significant increases (p < 0.001). - Participants with the least exposure obtained the greatest increases. - Significant increase in likelihood of target score (45/50) after completion (p < 0.001).	- Incomplete outcome data - Unable to tell whether intervention administered as intended
Easom et al. (2020), United States of America	To assess and evaluate dementia knowledge and self-efficacy in two cultural groups (Hispanic and Caucasian) prior to training participation and at the training end.	Pre-post intervention study	Community centers. Family carers (*n =* 671) - Caucasian, non-Hispanic (*n =* 567) - Hispanic (*n =* 104)	4 h workshop and take-home guide. Six sections: introduction, understanding dementia, general caregiving tips, dealing with behavioral issues, taking better care of yourself and resources.	Knowledge of AD (ADKS)	- Statistically significant increases Alzheimer's knowledge (all p < 0.001). - Caucasian ADKS mean difference: 3.64. - Hispanic ADKS mean difference: 2.86.	- Confounders not accounted for - Unable to tell whether intervention administered as intended
Noel et al. (2022), United States of America	To evaluate whether this virtual carer education program changes carer confidence, self-efficacy, and burden relative to controls.	Non-randomized experimental study	Virtual. Carers of people with dementia in Western North Carolina Region (*n =* 231) - Enrolled in course (*n =* 151) - Control (*n =* 80)	5 modules—what is dementia, transitioning from independence to interdependence, functional and behavioral changes of dementia, dementia treatment options and risk reduction, maintaining your own health.	Perceived increases in knowledge	−100% perceived knowledge increase (*n =* 87). - Non-significant increase between the virtual course and the in-person course (~97%) (p=0.08)	- Incomplete outcome data

^*^Outcomes and key findings only reported for outcomes of interest (knowledge of dementia/brain and learning experience).

### 3.2. Description of current interventions

For studies included in this review, the reported interventions did provide informal carers an education on the brain anatomy and physiology of people with dementia. None of the interventions focused solely on providing neurological education. Most studies were unclear in providing specific information surrounding the content contained within the intervention. The studies all provided brief outlines of the topic for each lesson or session; however, most did not provide any examples of the content, or information on the extent and depth of the anatomy and physiology information contained within.

### 3.3. Impact of interventions on knowledge

The six included studies used different measures to assess the impact of the presented educational intervention on dementia-specific knowledge. Four studies used validated scales to assess knowledge changes, with the most common being the Alzheimer's Disease Knowledge Scale (ADKS) (Hattink et al., 2015; Easom et al., 2020; van Wezel et al., 2021) and the other being the Dementia Knowledge Assessment Scale (DKAS) (Eccleston et al., 2019). Of the remaining studies, one utilized pre- and post-program surveys to report perceived increases in knowledge (Noel et al., 2022), while the other used 5-point Likert scales for participants to self-rate their knowledge pre- and post-intervention (Taylor-Rubin et al., 2020).

Five out of the six studies reported increases in knowledge following the educational intervention. Two of the studies using the ADKS reported significant increases in knowledge (all *p* < 0.001) (Easom et al., 2020; van Wezel et al., 2021). One of these studies also reported affect over time in intervention and control groups, with increased knowledge about dementia over time, reported in both conditions. At the three time points (T0, T1, T2), the intervention and control groups reported the respective scores of 7.6 (95% CI: 7.1–8.1), 9.0 (95% CI: 8.6–9.5), and 8.9 (95% CI: 8.4–9.4); 6.7 (95% CI: 6.2–7.2), 6.9 (95% CI: 6.5–7.4), and 7.4 (95% CI: 6.9–7.8). Over the span of the intervention, the intervention group had a significantly stronger (*p* < 0.001) knowledge increases compared to the control groups knowledge increase over time (van Wezel et al., 2021).

Using the DKAS validated scale, the study by Eccleston et al. (2019), utilized an online course to deliver dementia education globally. All groups in this study, regardless of exposure to dementia, previous dementia education, or general educational achievement, demonstrated increases in post-test scores, with the greatest increase being in people with the least exposure to dementia. Following completion of the course, the likelihood of achieving a target score (45/50) was significantly higher for all groups (*p* < 0.001).

Both studies using non-validated scales also reported increases in knowledge. The study by Noel et al. (2022), reported that 100% of participants (*n* = 87) reported perceived increases in their knowledge following completion of the virtual educational intervention, compared to a previous version of the program, delivered in-person, in which 97% (*n* = 88) of the participants reported perceived increases in knowledge. An intervention specifically targeting knowledge of primary progressive aphasia (PPA) in people with PPA (pwPPA) and their informal carers found significant increases in perceived knowledge following the intervention for all informal carers (first session (*p* < 0.01) and subsequent (*p* < 0.025)] and pwPPA attending their first session (Taylor-Rubin et al., 2020).

One study did not report any significant changes in dementia knowledge following the intervention using the ADKS. A randomized controlled trial of the STAR training portal reported no significant difference (*p* = 0.90) in pre- and post-test scores (Hattink et al., 2015).

### 3.4. Learning experience

The study by Hattink et al. (2015) also analyzed the learning experience and user satisfaction with the training portal. Overall, participants were positive about the usefulness of the modules and reported high user friendliness and usefulness across most elements. For laypeople, the relevant module to this review, module 1: “What is dementia?”, reported a mean usefulness of 8.07/10 in the Netherlands (*n* = 15) and 8.22/10 in the UK (*n* = 9). All elements except for the videos (2.0) were rated as 3.0 (useful) or higher for laypeople (*n* = 26) on a scale ranging from 1 to 4. Additionally, most laypeople completely agreed that all areas of the portal were user friendly.

### 3.5. Quality appraisal

All studies met the first two screening criteria for MMAT2018. Two studies were assessed using quantitative RCT appraisal criteria (Hattink et al., 2015; van Wezel et al., 2021), three using quantitative non-RCT criteria (Eccleston et al., 2019; Easom et al., 2020; Noel et al., 2022), and one using mixed-methods appraisal (Taylor-Rubin et al., 2020). None of these studies met all the quality appraisal criteria. One or more concerns were noted in these studies. All studies, except Easom et al. (2020), did not consist of complete outcome data with participants excluded from analysis, either due to missing data or reasons which were not reported (Hattink et al., 2015; Eccleston et al., 2019; Taylor-Rubin et al., 2020; van Wezel et al., 2021; Noel et al., 2022). This can lead to non-response bias during results interpretation. Secondly, the two studies assessed using the RCT quality appraisal criteria had concerns with blinding of outcome assessors (Hattink et al., 2015; van Wezel et al., 2021). This was primarily due to participants self-reporting knowledge, and due to the nature of the intervention, they were unable to be blinded. Third, two studies assessed using non-RCT quality appraisal criteria had issues with confounders not being accounted for in design and analysis (Easom et al., 2020; Taylor-Rubin et al., 2020). Finally, in two studies, it was not possible to determine whether the intervention was administered as intended (Eccleston et al., 2019; Easom et al., 2020).

## 4. Discussion

The aim of this review was to identify, appraise, and synthesize the existing evidence on the use of educational interventions to provide physiological and anatomical dementia education for informal carers of people with dementia and assess its effect on knowledge. It also sought to determine any opinions on the perceived usefulness and user friendliness of these interventions. All six articles included in this review provided a clear description of the evaluation process and implementation procedure; however, the exact educational content provided was not often presented reducing the usefulness or transferability of the findings to a broad audience. The studies were conducted across four countries with healthcare systems similar to Australia. The search was limited to the past 10 years (2012–2022) to ensure that the information presented in the intervention was current; however, from this sample, the oldest study was from 2015, demonstrating that the provision of dementia-specific anatomical and physiological education is a recent area of interest. All studies demonstrated some assessment of dementia knowledge to different extents, with both validated scales, perceived ratings and self-developed scales used to assess knowledge. Due to these varying methods, it is not possible to perform a meta-analysis of the results. Overall, the methodological quality was low, with blinding not possible and incomplete outcome data. Additionally, while all studies provided some component of anatomical and physiological education, no studies provided this as a sole intervention. Information surrounding additional factors in the disease process such as biomarkers involved in dementia (e.g., amyloid and tau protein accumulation in Alzheimer's disease) was not found to be included in the interventions; however, overall, the studies did not specifically state the exact content provided and due to this, the inclusion of this additional information may have occurred. As such, this makes it challenging to assess the sole effect of anatomical and physiological dementia education on knowledge. The small number of studies included in this review reflects the paucity of research in the field of physiological and anatomical dementia education for informal carers.

### 4.1. Overall effects of interventions on knowledge

While there is variability in the methods used to assess efficacy, most studies reported increases in knowledge. Of the three studies using the validated ADKS, two demonstrated significant increases in scores (Easom et al., 2020; van Wezel et al., 2021). The other study presented no significant increase between the experimental or control groups (Hattink et al., 2015). The two studies demonstrating statistically significant improvements were both delivered in-person, whereas the other was a virtual program. However, two other virtual programs, one using a validated scale (Eccleston et al., 2019) and one analyzing perceived knowledge (Noel et al., 2022), both demonstrated increases in knowledge. Additionally, the study by Noel et al. (2022) reported no significant differences in knowledge between the virtual program described in the study and an earlier-version delivered in person. The final study, delivered in-person, also reported increases in knowledge (Taylor-Rubin et al., 2020). As such, it is likely that both virtual and in-person programs can be utilized to deliver educational interventions for informal carers of people with dementia. While they have recently focused primarily on electronic based interventions, previous reviews and studies surrounding informational interventions for informal carers of people with dementia have also found that varying delivery methods, including telephone, computer and mixed-delivery based interventions, are suitable (Waller et al., 2017; Klimova et al., 2019; Pleasant et al., 2020; Naunton Morgan et al., 2022). Furthermore, the study reporting no significant changes, and unlike other included studies, did not require participants to complete all training modules. As part of this study, only one module delivered education on what dementia is and how it relates to the brain. As such, participants may not have completed this module, meaning that the intervention would not have affected physiological and anatomical-specific dementia knowledge. Additionally, this study grouped informal carers and volunteers during analysis, and as such, it is not possible to determine whether there was any effect on knowledge gains between the groups.

All three studies reporting significant (all p < 0.001) increases in measured knowledge specifically mentioned including information on the brain and how dementia relates to the brain. Some of the content specifically mentioned from these studies included “dementia is a brain disease”, “basic neurobiology and dementia pathophysiology”, and “basic information on how dementia affects the brain”. The results from these studies suggest that the provision of information surrounding the anatomy and physiology of the brain and dementia assists in significantly improving informal carers dementia knowledge.

Improved knowledge may lead to better outcomes for both informal carers and people with dementia. In particular, an improved understanding of the biomedical causes of dementia symptoms, including behavioral and psychological symptoms of dementia (BPSD), may alter informal carers attributions, leading to improved communication and quality of care (Polenick et al., 2018). Difficult behaviors can result in negative reactions, if the person with dementia is perceived to be responsible, leading to negative communication. This can result in amplifications of BPSD symptoms and result in long-term decreases in quality of care (Tarrier et al., 2002; Tynan and Allen, 2002; Polk, 2005; Kales et al., 2015; Chen et al., 2017; Polenick et al., 2018). Additionally, studies suggest that caregiver attributions affect their wellbeing, with higher care burden and elevated depression when symptoms are perceived to be personality traits or controllable (Tarrier et al., 2002; Martin-Cook et al., 2003; Williamson et al., 2005; Polenick and Martire, 2013; Polenick et al., 2018). While learning of a dementia diagnosis can assist caregivers to understand the behavioral changes observed in a person with dementia (Woods, 1995; Vernooij-Dassen et al., 2006), many people hold limited knowledge about the condition and the changes it can cause (Chung, 2000; Paton et al., 2004; Preston et al., 2007; Quinn et al., 2008). Therefore, by providing informal carers with information that explains the anatomical and physiological changes associated with dementia and their associated effects on behavior, it can lead to an improvement in attributions, with informal carers able to differentiate between the persons personality and pathological processes associated with dementia. These altered attributions can then lead to improved carer wellbeing and mental health, resulting in overall improvements in long-term care and outcomes.

### 4.2. Quality of assessment scales

The ADKS was the most common scale used in the included studies. While this is a validated scale, most questions focus on symptoms, risk factors and progression (Carpenter et al., 2009). As such, this scale may not be appropriate to assess the effect of interventions on knowledge surrounding the anatomy and physiology of dementia and the brain. Alternatively, the DKAS contains sections on causes and characteristics, which includes an area surrounding brain changes (Annear et al., 2017). Therefore, this scale may be more appropriate for assessments of knowledge surrounding the anatomy and physiology of dementia and the brain. Perceived assessment of knowledge provides insight into how useful participants found the interventions; however, it does not allow for analysis into the effects of the interventions on specific aspects of dementia knowledge. Future assessments and studies focusing on the impact of interventions on knowledge of the anatomy and physiology of dementia and the brain should seek to ensure that appropriate, validated scales, are utilized.

### 4.3. Other factors in interventions

When analyzing the results from the included studies, surrounding increases in knowledge, it is difficult to interpret the sole impact that anatomical and physiological dementia education has on either perceived knowledge increases or score increases from validated scales. This is due to the presence of additional educational or support factors present in all included studies. Common themes in additional education included caregiving skills, mental health and wellbeing support, and information about formal support resources. In addition to this other education, other components included the use of support groups and guided discussions, as well as take home guides for some of the in-person interventions. As some, or all, of these elements are present throughout all the included studies, it is difficult to fully ascertain the sole impact of the dementia education component. However, the promising results suggest that further research should be conducted to determine the efficacy of individual components.

### 4.4. Overall user experience

Only one study measured user experience. Participants using the STAR Training Portal were overall positive about the experience (Hattink et al., 2015). Whilst this intervention did not improve knowledge, the delivery method and online layout demonstrated high user friendliness and demonstrated that laypeople are willing to utilize interactive, virtual, and self-directed learning tools. Additionally, on average, layperson participants found the module targeting physiological and anatomical-specific dementia knowledge to be useful. While it did not directly analyze user friendliness and usefulness, the study delivering in-person PPA education and support groups reported overall positive qualitative themes regarding the delivery and content of the intervention following interviews with participants (Taylor-Rubin et al., 2020). Therefore, while further research is required on user experience in these interventions, the study outcomes indicate that informal carers of people with dementia are willing to use these interventions, whether virtual or in-person, and report a good user experience. The information provision should be tailored to the specific groups receiving the education, with factors such as technological literacy, previous education, and time-constraints influencing the method of delivery and content.

### 4.5. Strengths and limitations

To our knowledge, this is the first systematic review to examine current quantitative and qualitative evidence into the effect of educational interventions used to provide physiological and anatomical dementia education for informal carers of people with dementia on knowledge. The use of defined inclusion and exclusion criteria, rigorous search strategy of eight databases and validated MMAT2018 use for quality assessment are strengths of this review. Additionally, four of the six included studies utilized validated assessment scales to measure relevant outcomes. Five studies also included large participant numbers allowing for improved confidence in reported results.

As a limitation, the inclusion of quantitative non-randomized and mixed-methods studies does not allow for the establishment of efficacy, considering factors such as non-randomization or lack of causality, for all the studies. Language bias should also be considered as a limitation, as, in this review, only publications published in English were included. This may not consider studies published in other languages. The heterogeneity of the study designs, with regards to both the interventions and outcome assessment, made it unfeasible to conduct further analysis, such as meta-analysis of the included studies. Methodological quality of the included studies was low, with issues noted throughout all included studies. As such, further research is required demonstrating higher methodological quality in order to better ascertain the interventions effects on knowledge. However, the results from this review indicate that providing anatomical and physiological specific education to informal carers in clinical practice is effective at improving knowledge of dementia. While further research is needed, it is possible that this increased knowledge may lead to improved outcomes, carer wellbeing, and quality of care.

This study did not incorporate results surrounding changes in attitude or attributions following the use of an educational intervention. However, education has been suggested to affect these, with a combination of increased knowledge and understanding and improved attitudes and attributions potentially leading to better outcomes, informal carer wellbeing and better care. As such, further research incorporating studies assessing knowledge, attitudes and attributions should be conducted. Additionally, future research should work on developing educational interventions on brain anatomy and physiology which can be delivered to people with dementia and their informal carers in order to improve understanding, and communication with healthcare professionals during the dementia diagnosis and management process. Further research should also be conducted as to whether this education should be provided as a stand-alone program, as part of the diagnostic process, or provided in addition to current resources and programs. Additionally, studies could also focus on the provision of education to individuals diagnosed with mild cognitive impairment (MCI) in order to improve early diagnosis and treatment if there is a progression to dementia.

### 4.6. Conclusions

Currently, most dementia educational interventions are focused on caregiving skills and carer support. However, educational interventions focusing on physiological and anatomical dementia education demonstrate an important area of knowledge for informal dementia carers. While there are limited studies in this space, and despite the low methodological quality, most studies identified in this review reported increased dementia knowledge. Regardless of whether the intervention was delivered face-to-face or virtually, most methods demonstrated the ability to improve dementia knowledge. As such, these interventions can be formulated to be delivered in varied settings allowing application across a wide range of environments and situations. The reports of high user friendliness and usefulness also show that these interventions are well received by informal carers of people with dementia. As a WHO priority for dementia management, there is a surprising paucity of research into the provision of dementia education. Studies do not always provide details of content presented, nor assess the benefits to dementia management from the provision of information. In particular, with a clear comprehension of dementia dependent on at least some understanding of brain anatomy and physiology, there is an identified need to incorporate this into current resources provided to dementia patients and their families.

## Data availability statement

The original contributions presented in the study are included in the article/Supplementary material, further inquiries can be directed to the corresponding author.

## Author contributions

DB contributed to validation, investigation, writing of the original draft, and subsequent edits. CJ and CM additionally contributed to supervision and review. All authors contributed to conceptualization, methodology, formal analysis, editing, and visualization.

## References

[B1] Annear M. J. Toye C. Elliott K.-E. J. McInerney F. Eccleston C. Robinson A. (2017). Dementia knowledge assessment scale (DKAS): confirmatory factor analysis and comparative subscale scores among an international cohort. BMC Geriatr. 17, 168. 10.1186/s12877-017-0552-y 28760154 PMC5537989

[B2] Barrett A. M. Orange W. Keller M. Damgaard P. Swerdlow R. H. (2006). Short-term effect of dementia disclosure: how patients and families describe the diagnosis. J. Am. Geriatr. Soc. 54, 1968–1970. 10.1111/j.1532-5415.2006.00992.x 17198527

[B3] Carpenter B. D. Balsis S. Otilingam P. G. Hanson P. K. Gatz M. (2009). The Alzheimer's Disease Knowledge Scale: development and psychometric properties. Gerontologist. 49, 236–247. 10.1093/geront/gnp023 19363018 PMC2667675

[B4] Chen C. K. Clayton K. Chodosh J. (2017). The relationship between “what we believe” and “how we care” among daughters caring for a parent with dementia. Am. J. Alzheimers. Dis. Other Demen. 32, 90–95. 10.1177/1533317517689875 28116927 PMC10852901

[B5] Cheng S.-T. Zhang F. (2020). A comprehensive meta-review of systematic reviews and meta-analyses on nonpharmacological interventions for informal dementia caregivers. BMC Geriatr. 20, 137. 10.1186/s12877-020-01547-2 32293325 PMC7158025

[B6] Chung J. C. (2000). Lay interpretation of dementia. Int. Psychogeriatr. 12, 369–377. 10.1017/S1041610200006475 11081957

[B7] Clark J. M. Sanders S. Carter M. Honeyman D. Cleo G. Auld Y. . (2020). Improving the translation of search strategies using the Polyglot Search Translator: a randomized controlled trial. J. Med. Libr. Assoc. 108, 195–207. 10.5195/jmla.2020.834 32256231 PMC7069833

[B8] Cunningham E. L. McGuinness B. Herron B. Passmore A. P. (2015). Dementia. Ulster Med. J. 84, 79−87. PMC4488926 26170481

[B9] Duong S. Patel T. Chang F. (2017). Dementia: what pharmacists need to know. Can Pharm J (Ott) 150, 118–129. 10.1177/1715163517690745 28405256 PMC5384525

[B10] Easom L. Wang K. Alston G. (2020). Increasing self-efficacy and knowledge in carer training: Hispanic vs. Caucasian. Nurs Open 7, 180–185. 10.1002/nop2.376 31871701 PMC6917978

[B11] Eccleston C. Doherty K. Bindoff A. Robinson A. Vickers J. McInerney F. (2019). Building dementia knowledge globally through the understanding dementia massive open online course (MOOC). NPJ Sci. Learn. 4, 3. 10.1038/s41539-019-0042-4 30993003 PMC6458180

[B12] Hattink B. Meiland F. van der Roest H. Kevern P. Abiuso F. Bengtsson J. . (2015). Web-based STAR E-learning course increases empathy and understanding in dementia caregivers: results from a randomized controlled trial in the Netherlands and the United Kingdom. J. Med. Internet Res. 17, e241. 10.2196/jmir.4025 26519106 PMC4642792

[B13] Hildreth K. L. Church S. (2015). Evaluation and management of the elderly patient presenting with cognitive complaints. Med. Clin. North Am. 99, 311–335. 10.1016/j.mcna.2014.11.006 25700586 PMC4399854

[B14] Hong Q. N. Fàbregues S. Bartlett G. Boardman F. Cargo M. Dagenais P. . (2018). The Mixed Methods Appraisal Tool (MMAT) version 2018 for information professionals and researchers. Educ. Inform. 34, 1–7. 10.3233/EFI-18022129132909

[B15] James H. J. Van Houtven C. H. Lippmann S. Burke J. R. Shepherd-Banigan M. Belanger E. . (2020). How accurately do patients and their care partners report results of amyloid-β pet scans for alzheimer's disease assessment? J. Alzheimers. Dis. 74, 625–636. 10.3233/JAD-190922 32065790 PMC7183243

[B16] Kales H. C. Gitlin L. N. Lyketsos C. G. (2015). Assessment and management of behavioral and psychological symptoms of dementia. BMJ. 350, h369. 10.1136/bmj.h369 25731881 PMC4707529

[B17] Khanassov V. Rojas-Rozo L. Sourial R. Yang X. Q. Vedel I. (2021). Needs of patients with dementia and their caregivers in primary care: lessons learned from the Alzheimer plan of Quebec. BMC Fam. Pract. 22, 186. 10.1186/s12875-021-01528-3 34525960 PMC8441033

[B18] Klimova B. Valis M. Kuca K. Masopust J. (2019). E-learning as valuable caregivers' support for people with dementia - A systematic review. BMC Health Serv. Res. 19, 781. 10.1186/s12913-019-4641-9 31676005 PMC6824008

[B19] Kurz A. Schulz M. Reed P. Wortmann M. Rodrigo J. Hohlbein H. . (2008). Personal perspectives of persons with Alzheimer's disease and their carers: A global survey. Alzheimers Dement. 4, 345–352. 10.1016/j.jalz.2008.06.002 18790461

[B20] Lee S. Y. Arozullah A. M. Cho Y. I. (2004). Health literacy, social support, and health: a research agenda. Soc. Sci. Med. 58, 1309–1321. 10.1016/S0277-9536(03)00329-0 14759678

[B21] Martin-Cook K. Remakel-Davis B. Svetlik D. Hynan L. S. Weiner M. F. (2003). Caregiver attribution and resentment in dementia care. American Journal of Alzheimer's Disease & Other Dementias^®^ 18, 366–374. 10.1177/153331750301800606 14682086 PMC10833658

[B22] McKhann G. M. Knopman D. S. Chertkow H. Hyman B. T. Jack C. R. Jr. Kawas C. H. . (2011). The diagnosis of dementia due to Alzheimer's disease: recommendations from the National Institute on Aging-Alzheimer's Association workgroups on diagnostic guidelines for Alzheimer's disease. Alzheimers. Dement. 7, 263–269. 10.1016/j.jalz.2011.03.005 21514250 PMC3312024

[B23] Mittelman M. S. Haley W. E. Clay O. J. Roth D. L. (2006). Improving caregiver well-being delays nursing home placement of patients with Alzheimer disease. Neurology. 67, 1592–1599. 10.1212/01.wnl.0000242727.81172.91 17101889

[B24] Molnar F. Frank C. C. (2018). Support of caregivers of persons with dementia. Can. Fam. Physician 64, 294. PMC5897072 29650606

[B25] Naunton Morgan B. Windle G. Sharp R. Lamers C. (2022). eHealth and web-based interventions for informal carers of people with dementia in the community: Umbrella review. J. Med. Internet Res. 24, e36727. 10.2196/36727 35867388 PMC9356334

[B26] Noel M. A. Lackey E. Labi V. Bouldin E. D. (2022). Efficacy of a virtual education program for family caregivers of persons living with dementia. J. Alzheimers. Dis. 86, 1667–1678. 10.3233/JAD-215359 35213371 PMC9108574

[B27] Page M. J. McKenzie J. E. Bossuyt P. M. Boutron I. Hoffmann T. C. Mulrow C. D. . (2021). The PRISMA 2020 statement: an updated guideline for reporting systematic reviews. BMJ 372, n71. 10.1136/bmj.n71 33782057 PMC8005924

[B28] Paton J. Johnston K. Katona C. Livingston G. (2004). What causes problems in Alzheimer's disease: attributions by caregivers. A qualitative study. Int. J. Geriatr Psychiatry. 19, 527–532. 10.1002/gps.1118 15211530

[B29] Peterson K. Hahn H. Lee A. J. Madison C. A. Atri A. (2016). In the information age, do dementia caregivers get the information they need? Semi-structured interviews to determine informal caregivers' education needs, barriers, and preferences. BMC Geriatrics. 16, 164. 10.1186/s12877-016-0338-7 27662829 PMC5035467

[B30] Pleasant M. Molinari V. Dobbs D. Meng H. Hyer K. (2020). Effectiveness of online dementia caregivers training programs: A systematic review. Geriatr. Nurs. 41, 921–935. 10.1016/j.gerinurse.2020.07.004 32703628

[B31] Polenick C. A. Martire L. M. (2013). Caregiver attributions for late-life depression and their associations with caregiver burden. Fam. Process. 52, 709–722. 10.1111/famp.12032 24329412 PMC4761441

[B32] Polenick C. A. Struble L. M. Stanislawski B. Turnwald M. Broderick B. Gitlin L. N. . (2018). “The filter is kind of broken”: family caregivers' attributions about behavioral and psychological symptoms of dementia. Am. J. Geriatr. Psychiatry. 26, 548–556. 10.1016/j.jagp.2017.12.004 29373300 PMC6619504

[B33] Polk D. M. (2005). Communication and family caregiving for Alzheimer's dementia: linking attributions and problematic integration. Health Commun. 18, 257–273. 10.1207/s15327027hc1803_4 16187931

[B34] Preston L. Marshall A. Bucks R. S. (2007). Investigating the ways that older people cope with dementia: a qualitative study. Aging Ment. Health. 11, 131–143. 10.1080/13607860600844572 17453546

[B35] Quinn C. Clare L. Pearce A. van Dijkhuizen M. (2008). The experience of providing care in the early stages of dementia: an interpretative phenomenological analysis. Aging Ment. Health 12, 769–778. 10.1080/13607860802380623 19023728

[B36] Robinson A. Elder J. Emden C. Lea E. Turner P. Vickers J. (2009). Information pathways into dementia care services: family carers have their say. Dementia. 8, 17–37. 10.1177/1471301208099051

[B37] Savva G. M. Wharton S. B. Ince P. G. Forster G. Matthews F. E. Brayne C. (2009). Age, neuropathology, and dementia. N. Engl. J. Med. 360, 2302–2309. 10.1056/NEJMoa0806142 19474427

[B38] Tarrier N. Barrowclough C. Ward J. Donaldson C. Burns A. Gregg L. (2002). Expressed emotion and attributions in the carers of patients with Alzheimer's disease: the effect on carer burden. J. Abnorm. Psychol. 111, 340–349. 10.1037/0021-843X.111.2.340 12003455

[B39] Taylor-Rubin C. Azizi L. Croot K. Nickels L. (2020). Primary progressive aphasia education and support groups: a clinical evaluation. Am. J. Alzheimer's Dis. Other Dement. 35, 1533317519895638. 10.1177/1533317519895638 32096652 PMC10691435

[B40] Tynan H. Allen D. (2002). The impact of service user cognitive level on carer attributions for aggressive behaviour. J. Appl. Res. Intellect. Disabil. 15, 213–223. 10.1046/j.1468-3148.2002.00120.x

[B41] van Wezel N. van der Heide I. Devill,é W. L. J. M. Kayan Acun E. Meerveld J. H. C. M. Spreeuwenberg P. . (2021). Effects of an educational peer-group intervention on knowledge about dementia among family caregivers with a Turkish or Moroccan immigrant background: a cluster randomised controlled trial. Patient Educ. Couns. 104, 1726–1735. 10.1016/j.pec.2020.11.008 33279344

[B42] Vernooij-Dassen M. Derksen E. Scheltens P. Moniz-Cook E. (2006). Receiving a diagnosis of dementia: the experience over time. Dementia 5, 397–410. 10.1177/1471301206067114

[B43] Waller A. Dilworth S. Mansfield E. Sanson-Fisher R. (2017). Computer and telephone delivered interventions to support caregivers of people with dementia: a systematic review of research output and quality. BMC Geriatr. 17, 265. 10.1186/s12877-017-0654-6 29145806 PMC5691399

[B44] Wesson V. Reitman J. S. (2012). Refining Dementia Intervention: The Caregiver-Patient Dyad as the Unit of Care. Canadian Geriatrics Society journal of CME.

[B45] Whittemore R. Knafl K. (2005). The integrative review: updated methodology. J. Adv. Nurs. 52, 546–553. 10.1111/j.1365-2648.2005.03621.x 16268861

[B46] Williamson G. M. Martin-Cook K. Weiner M. F. Svetlik D. A. Saine K. Hynan L. S. . (2005). Caregiver resentment: explaining why care recipients exhibit problem behavior. Rehabil. Psychol. 50, 215–223. 10.1037/0090-5550.50.3.215

[B47] Woods B. O. B. (1995). Dementia care: progress and prospects. J. Mental Health. 4, 115–124. 10.1080/09638239550037659

[B48] World Health Organisation (2017). Global Action Plan on the Public Health Response to Dementia 2017.

[B49] World Health Organisation (2022). Dementia. Available online at: https://www.who.int/news-room/fact-sheets/detail/dementia (accessed January 2, 2023).

[B50] Zwingmann I. Michalowsky B. Esser A. Kaczynski A. Monsees J. Keller A. . (2019). Identifying unmet needs of family dementia caregivers: results of the baseline assessment of a cluster-randomized controlled intervention trial. J Alzheimers Dis. 67, 527–539. 10.3233/JAD-180244 30584136 PMC6398541

